# Bis[2,6-bis­(4,5-dihydro-1*H*-imidazol-2-yl)pyridine]manganese(II) bis­(per­chlorate) acetonitrile solvate

**DOI:** 10.1107/S1600536809029195

**Published:** 2009-08-08

**Authors:** Shao-Ming Shang, Chun-Xia Ren, Xin Wang, Lu-De Lu, Xu-Jie Yang

**Affiliations:** a School of Chemical and Materials Engineering, Nanjing University of Science and Technology, 200 Xiaolingwei Road, Nanjing, Jiangsu Province 210094, People’s Republic of China; b School of Chemical and Materials Engineering, Jiangnan University, 1800 Lihu Road, Wuxi, Jiangsu Province 214122, People’s Republic of China

## Abstract

In the cation of the title compound, [Mn(C_11_H_13_N_5_)_2_](ClO_4_)_2_·CH_3_CN, the metal atom is located on a twofold rotation axis and is six-coordinated by six N atoms from two different 2,6-bis­(4,5-dihydro-1*H*-imidazol-2-yl)pyridine (bip) ligands in a distorted octahedral geometry. The O atoms of the perchlorate anions are disordered with occupancies in the ratio 0.593 (10):0.407 (10). In the crystal, mol­ecules are stabilized by two N—H⋯O hydrogen bonds, forming zigzag chains along the *a* axis, which are further inter­connected by N—H⋯O hydrogen bonds and π–π inter­actions [centroid–centroid distance = 3.50 (1) Å] into a three-dimensional network.

## Related literature

For the network topologies and potential applications of supra­molecular architectures, see: Yaghi *et al.* (1998 ▶); Hagrman *et al.* (1999 ▶). The protonation and deprotonation of an imidazole ligand is believed to play an important role in the mechanism of the coordination chemistry, see: Bordo *et al.* (2001 ▶). Our studies of such complexes involving an imidazole ligand indicate that hydrogen bonding involving this group influences the geometry around the metal atom and the crystallization mechanism, see: Ren *et al.* (2007 ▶, 2009 ▶); Ren, Ye, He *et al.* (2004 ▶); Ren, Ye, Zhu *et al.* (2004 ▶). For metal–imidazole bond lengths, see: Stupka *et al.* (2004 ▶); Hammes *et al.* (2005 ▶); Haga *et al.* (1996 ▶); Böca *et al.* (2005 ▶). For metal–imidazole bond lengths, see: Ren *et al.* (2009 ▶). For the synthesis of 2,6-bis­(4,5-dihydro-1*H*-imidazol-2-yl)pyridine, see: Baker *et al.* (1991 ▶). 
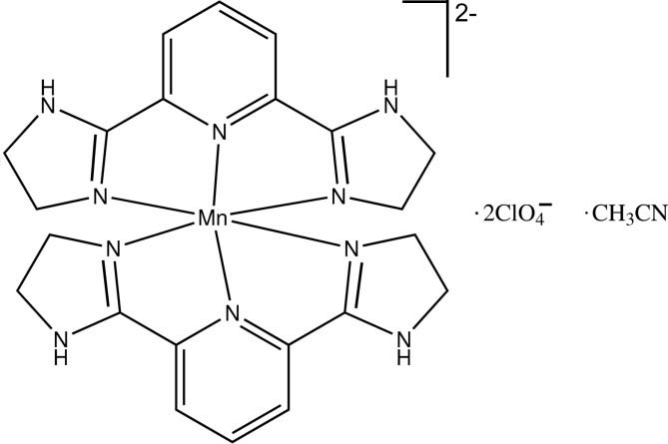


## Experimental

### 

#### Crystal data

[Mn(C_11_H_13_N_5_)_2_](ClO_4_)_2_·C_2_H_3_N
*M* *_r_* = 725.42
Monoclinic, 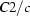

*a* = 20.521 (5) Å
*b* = 12.732 (5) Å
*c* = 14.602 (6) Å
β = 123.893 (10)°
*V* = 3167.0 (19) Å^3^
*Z* = 4
Mo *K*α radiation
μ = 0.65 mm^−1^
*T* = 273 K
0.28 × 0.21 × 0.14 mm

#### Data collection

Bruker SMART CCD area-detector diffractometer
Absorption correction: multi-scan (*SADABS*; Bruker, 1998 ▶) *T*_min_ = 0.837, *T*_max_ = 0.912
7799 measured reflections
2821 independent reflections
1277 reflections with *I* > 2σ(*I*)
*R*_int_ = 0.056

#### Refinement

*R*[*F* ^2^ > 2σ(*F* ^2^)] = 0.047
*wR*(*F* ^2^) = 0.121
*S* = 0.79
2821 reflections
246 parameters
94 restraints
H-atom parameters constrained
Δρ_max_ = 0.33 e Å^−3^
Δρ_min_ = −0.27 e Å^−3^

### 

Data collection: *SMART* (Bruker, 1998 ▶); cell refinement: *SAINT-Plus* (Bruker, 1998 ▶); data reduction: *SAINT-Plus*; program(s) used to solve structure: *SHELXTL* (Sheldrick, 2008 ▶); program(s) used to refine structure: *SHELXTL*; molecular graphics: *SHELXTL*; software used to prepare material for publication: *SHELXTL*.

## Supplementary Material

Crystal structure: contains datablocks global, I. DOI: 10.1107/S1600536809029195/tk2501sup1.cif

Structure factors: contains datablocks I. DOI: 10.1107/S1600536809029195/tk2501Isup2.hkl

Additional supplementary materials:  crystallographic information; 3D view; checkCIF report

## Figures and Tables

**Table 1:** Selected geometric parameters (Å, °)

Mn1—N4	2.247 (3)
Mn1—N2	2.283 (3)
Mn1—N1	2.287 (3)

**Table 2:** Hydrogen-bond geometry (Å, °)

*D*—H⋯*A*	*D*—H	H⋯*A*	*D*⋯*A*	*D*—H⋯*A*
N5—H5*A*⋯O2^ii^	0.86	2.50	3.237 (12)	144
N5—H5*A*⋯O3^ii^	0.86	2.25	2.942 (12)	137
N5—H5*A*⋯O2′^ii^	0.86	2.11	2.965 (8)	176
N3—H3*A*⋯O4^iii^	0.86	2.52	3.26 (2)	144
N3—H3*A*⋯O3′^iii^	0.86	2.16	3.015 (8)	178

Symmetry codes: (ii) 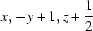; (iii) 
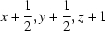
.

**Fig. 1.**
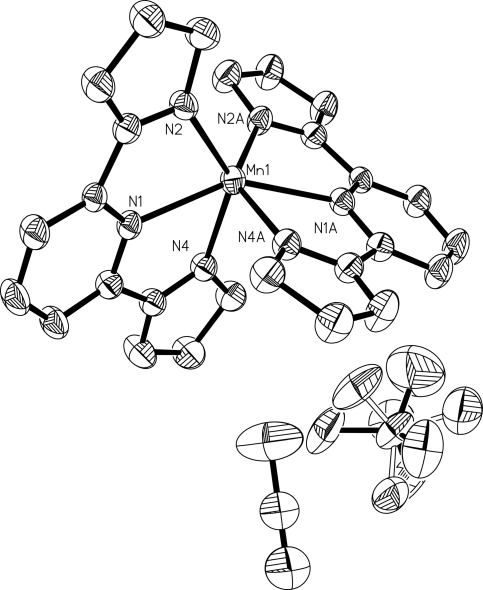
The structure of the complex [Mn(bip)2]2+ showing 30% probability displacement ellipsoids and H atoms have been omitted for clarity.

**Fig. 2.**
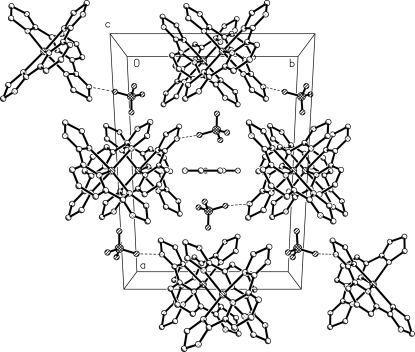
The framework of [Mn(bip)2](ClO4)2CH3CN viewed along the c axis. H atoms have been omitted for clarity.

## supplementary crystallographic information

### Comment

The construction supramolecular architectures is currently of great interest owing to their intriguing network topologies and potential functions such as adsorption, ion exchange, shape-selective catalysis, non-linear and magnetic materials (Yaghi *et al.*, 1998; Hagrman *et al.*, 1999). The protonation and deprotonation of an imidazole ligand is believed to play an important role in the mechanism of the coordination chemistry (Bordo, *et al.*, 2001). We described previously a number of such metal complexes, including imidazole ligand, and have concluded that hydrogen bonding involving this group influences the geometry around the metal atom and the crystallization mechanism (Ren, Ye, He *et al.*, 2004; Ren, Ye, Zhu *et al.*, 2004; Ren *et al.*, 2007, 2009). We report here the preparation and crystal structure of a mononuclear coordination complex, [Mn(bip)_2_](ClO_4_)_2_.CH_3_CN (I) (bip is 2,6-bis(4,5-dihydro-1*H*-imidazol-2-yl)pyridine).

The crystal structure of (I) crystallizes in the monoclinic space group *C*2/*c*. As shown in Fig. 1, the title compound consists of a [Mn(bip)_2_]^2+^ cation, two perchlorate counter ions and one Acetonitride molecular. The manganese(II) atom in the cation is in a distorted tetrahedral geometry, being coordinated with six nitrogen atoms from two neutral tridentate ligands bip. The Mn(1)—N bond lengths of The equatorial 2.292 (4), 2.284 (4), 2.251 (4) Å, which are slightly shorter than the metal-imidazole (Stupka, *et al.*, 2004; Hammes *et al.*, 2005; Haga *et al.*, 1996; Böca *et al.*, 2005) and longer than the metal-imidazole (Ren, *et al.*, 2009). The N—Mn(1)—N bond angle is in the range of 70.17 (15)–147.1 (2) /%. Two bip ligands of adjacent molecules are parallel to each other with a distance of 3.50 Å, showing the presence of π-π interaction. The molecules further interconnected into three-dimensional network through hydrogen bond between the oxygen atom of perchlorate counter-ion and the uncoordination nitrogen atoms of bip ligands.

### Experimental

All the reagents and solvents employed were commercially available and used as received without further purification. The ligand 2,6-bis(4,5-dihydro-1*H*-imidazol-2-yl)pyridine (bip) was synthesized by literature methods (Baker *et al.*, 1991).

A solution of MnCl_2_4H_2_O (0.2 mmol, 40 mg) and NaClO_4_ (0.4 mmol, 50 mg) in acetonitrile (10 ml), was added dropwise to a stirred solution of bip (0.4 mmol, 86 mg) in methanol(10 ml) at 60 K. Yellow single crystals suitable for X-ray diffraction were obtained by slow diffusion of diethyl ether into the clear filtrate for two days in 60% yield. Elemental analysis, Found: C, 39.68; H, 3.93; N, 21.15%. Calc. for C_24_H_29_C_l2_MnN_11_O_8_: C, 39.70; H, 4.00; N, 21.23%. Main IR bands (KBr, cm_-1_): 3370*m*, 3204 s, 2938*m*, 2887*m*, 1595*m*, 1567 s, 1531 s, 1453 s, 1283 s, 1209w, 1144 s,1116 s, 1089 s, 1028w, 1010*m*, 953w, 830w, 752w, 663w, 636*m*, 628*m*.

### Refinement

The H atom attached to N(2) atom was refined isotropically. All the other H atoms were placed in geometrically idealized positions and constrained to ride on their parent atoms with N—H and C—H distances of 0.90 Å and 0.96 Å, respectively, and *U*_iso_(H) = 1.2 times of those of their parent atoms (Å^2^). The O atoms are resolved into two positions by PART instructions. The occupancy for the unprimed O atoms is set at 21 and that of the primed atoms at -21. The clorine-oxygen distances were restrained to 1.44 Å (and the oxygen-oxygen interaction to 2.35 Å). Additionally, the vibration of the oxygen atoms were made isotropic by an ISOR restraint. The O atoms are resolved into two positions and give the site occupany of the major component.

### Crystal data

**Table:**

[Mn(C_11_H_13_N_5_)_2_](ClO_4_)_2_·C_2_H_3_N	*F*(000) = 1492
*M**_r_* = 725.42	*D*_x_ = 1.521 Mg m^−^^3^
Monoclinic, *C*2/*c*	Mo *K*α radiation, λ = 0.71073 Å
*a* = 20.521 (5) Å	Cell parameters from 668 reflections
*b* = 12.732 (5) Å	θ = 2.0–25.1°
*c* = 14.602 (6) Å	µ = 0.65 mm^−^^1^
β = 123.893 (10)°	*T* = 273 K
*V* = 3167.0 (19) Å^3^	Block, yellow
*Z* = 4	0.28 × 0.21 × 0.14 mm

### Data collection

**Table:**

Bruker SMART CCD area-detector diffractometer	2821 independent reflections
Radiation source: fine-focus sealed tube	1277 reflections with *I* > 2σ(*I*)
graphite	*R*_int_ = 0.056
φ and ω scans	θ_max_ = 25.1°, θ_min_ = 2.0°
Absorption correction: multi-scan (*SADABS*; Bruker, 1998)	*h* = −22→24
*T*_min_ = 0.837, *T*_max_ = 0.912	*k* = −11→15
7799 measured reflections	*l* = −16→17

### Refinement

**Table:**

Refinement on *F*^2^	Primary atom site location: structure-invariant direct methods
Least-squares matrix: full	Secondary atom site location: difference Fourier map
*R*[*F*^2^ > 2σ(*F*^2^)] = 0.047	Hydrogen site location: inferred from neighbouring sites
*wR*(*F*^2^) = 0.121	H-atom parameters constrained
*S* = 0.79	*w* = 1/[σ^2^(*F*_o_^2^) + (0.0647*P*)^2^] where *P* = (*F*_o_^2^ + 2*F*_c_^2^)/3
2821 reflections	(Δ/σ)_max_ = 0.001
246 parameters	Δρ_max_ = 0.33 e Å^−^^3^
94 restraints	Δρ_min_ = −0.27 e Å^−^^3^

### Special details

**Table:**

Geometry. All e.s.d.'s (except the e.s.d. in the dihedral angle between two l.s. planes) are estimated using the full covariance matrix. The cell e.s.d.'s are taken into account individually in the estimation of e.s.d.'s in distances, angles and torsion angles; correlations between e.s.d.'s in cell parameters are only used when they are defined by crystal symmetry. An approximate (isotropic) treatment of cell e.s.d.'s is used for estimating e.s.d.'s involving l.s. planes.
Refinement. Refinement of *F*^2^ against ALL reflections. The weighted *R*-factor *wR* and goodness of fit *S* are based on *F*^2^, conventional *R*-factors *R* are based on *F*, with *F* set to zero for negative *F*^2^. The threshold expression of *F*^2^ > σ(*F*^2^) is used only for calculating *R*-factors(gt) *etc*. and is not relevant to the choice of reflections for refinement. *R*-factors based on *F*^2^ are statistically about twice as large as those based on *F*, and *R*- factors based on ALL data will be even larger.

### Fractional atomic coordinates and isotropic or equivalent isotropic displacement parameters (Å^2^)

**Table:**

	*x*	*y*	*z*	*U*_iso_*/*U*_eq_	Occ. (<1)
Mn1	0.5000	0.94417 (7)	0.7500	0.0488 (3)	
N1	0.51856 (17)	0.8931 (3)	0.9132 (2)	0.0468 (8)	
N2	0.59227 (18)	1.0487 (2)	0.8898 (2)	0.0539 (8)	
N3	0.6779 (2)	1.0731 (3)	1.0725 (3)	0.0708 (10)	
H3A	0.6979	1.0635	1.1416	0.085*	
N4	0.40797 (17)	0.8262 (2)	0.7177 (2)	0.0524 (8)	
N5	0.3602 (2)	0.7083 (3)	0.7785 (3)	0.0833 (12)	
H5A	0.3581	0.6730	0.8271	0.100*	
C1	0.6436 (2)	1.1375 (4)	0.9029 (3)	0.0746 (13)	
H1A	0.6130	1.2013	0.8721	0.090*	
H1B	0.6693	1.1225	0.8652	0.090*	
C2	0.7041 (3)	1.1503 (4)	1.0259 (3)	0.0785 (14)	
H2A	0.7566	1.1347	1.0453	0.094*	
H2B	0.7032	1.2208	1.0504	0.094*	
C3	0.6168 (2)	1.0191 (3)	0.9886 (3)	0.0509 (10)	
C4	0.5788 (2)	0.9317 (3)	1.0073 (3)	0.0450 (9)	
C5	0.6011 (2)	0.8891 (3)	1.1076 (3)	0.0630 (12)	
H5	0.6430	0.9169	1.1733	0.076*	
C6	0.5594 (3)	0.8037 (4)	1.1076 (3)	0.0757 (14)	
H6	0.5736	0.7730	1.1741	0.091*	
C7	0.4970 (2)	0.7637 (4)	1.0097 (3)	0.0678 (13)	
H7	0.4689	0.7062	1.0090	0.081*	
C8	0.4778 (2)	0.8114 (3)	0.9136 (3)	0.0506 (10)	
C9	0.4140 (2)	0.7806 (3)	0.8007 (3)	0.0525 (11)	
C10	0.3053 (3)	0.6981 (4)	0.6593 (3)	0.0818 (14)	
H10A	0.2523	0.7166	0.6361	0.098*	
H10B	0.3054	0.6279	0.6335	0.098*	
N6	0.5000	0.3462 (7)	0.7500	0.186 (4)	
C12	0.5000	0.5403 (7)	0.7500	0.178 (5)	
H12B	0.5294	0.5712	0.7238	0.214*	0.50
H12A	0.5189	0.5583	0.8247	0.214*	0.50
H12C	0.4453	0.5583	0.7073	0.214*	0.50
C13	0.5000	0.4339 (7)	0.7500	0.116 (3)	
C11	0.3378 (2)	0.7797 (3)	0.6178 (3)	0.0664 (12)	
H11A	0.3520	0.7454	0.5720	0.080*	
H11B	0.2991	0.8334	0.5742	0.080*	
Cl1	0.32690 (9)	0.52285 (13)	0.39585 (12)	0.1026 (5)	
O1	0.3119 (7)	0.5391 (11)	0.4824 (8)	0.147 (6)	0.407 (10)
O2	0.4047 (4)	0.4898 (11)	0.4424 (8)	0.152 (7)	0.407 (10)
O3	0.2745 (6)	0.4409 (11)	0.3284 (13)	0.209 (9)	0.407 (10)
O4	0.3090 (11)	0.6156 (10)	0.3332 (15)	0.264 (11)	0.407 (10)
O1'	0.3558 (5)	0.6031 (6)	0.4718 (7)	0.151 (5)	0.593 (10)
O2'	0.3455 (6)	0.4194 (5)	0.4361 (7)	0.139 (5)	0.593 (10)
O3'	0.2464 (3)	0.5354 (7)	0.3137 (5)	0.139 (4)	0.593 (10)
O4'	0.3628 (7)	0.5401 (8)	0.3310 (10)	0.217 (7)	0.593 (10)

### Atomic displacement parameters (Å^2^)

**Table:**

	*U*^11^	*U*^22^	*U*^33^	*U*^12^	*U*^13^	*U*^23^
Mn1	0.0517 (6)	0.0556 (6)	0.0338 (5)	0.000	0.0205 (4)	0.000
N1	0.047 (2)	0.054 (2)	0.0324 (18)	−0.0002 (17)	0.0180 (17)	−0.0028 (15)
N2	0.059 (2)	0.058 (2)	0.0378 (19)	−0.0088 (18)	0.0229 (16)	0.0011 (16)
N3	0.074 (2)	0.084 (3)	0.0357 (19)	−0.030 (2)	0.0196 (19)	−0.0089 (19)
N4	0.051 (2)	0.059 (2)	0.0362 (18)	−0.0072 (17)	0.0177 (17)	−0.0039 (16)
N5	0.091 (3)	0.093 (3)	0.046 (2)	−0.046 (3)	0.027 (2)	−0.007 (2)
C1	0.088 (3)	0.072 (3)	0.054 (3)	−0.023 (3)	0.034 (3)	−0.004 (2)
C2	0.081 (3)	0.091 (4)	0.053 (3)	−0.030 (3)	0.032 (3)	−0.009 (2)
C3	0.049 (3)	0.055 (3)	0.042 (2)	−0.002 (2)	0.022 (2)	−0.004 (2)
C4	0.047 (2)	0.051 (3)	0.034 (2)	0.000 (2)	0.020 (2)	−0.002 (2)
C5	0.065 (3)	0.076 (3)	0.035 (2)	−0.010 (3)	0.019 (2)	−0.003 (2)
C6	0.093 (4)	0.089 (4)	0.039 (3)	−0.014 (3)	0.033 (3)	0.008 (2)
C7	0.080 (3)	0.076 (3)	0.040 (3)	−0.022 (3)	0.029 (2)	−0.001 (2)
C8	0.047 (2)	0.056 (3)	0.043 (2)	−0.005 (2)	0.021 (2)	−0.003 (2)
C9	0.054 (3)	0.055 (3)	0.040 (2)	−0.008 (2)	0.021 (2)	−0.003 (2)
C10	0.081 (3)	0.087 (4)	0.058 (3)	−0.034 (3)	0.027 (3)	−0.015 (3)
N6	0.304 (12)	0.120 (8)	0.196 (9)	0.000	0.179 (9)	0.000
C12	0.163 (10)	0.098 (8)	0.154 (9)	0.000	0.013 (7)	0.000
C13	0.153 (8)	0.094 (8)	0.124 (7)	0.000	0.091 (6)	0.000
C11	0.062 (3)	0.073 (3)	0.044 (2)	−0.018 (2)	0.017 (2)	−0.010 (2)
Cl1	0.0956 (11)	0.0967 (12)	0.0876 (10)	0.0154 (9)	0.0339 (9)	−0.0296 (9)
O1	0.165 (12)	0.155 (13)	0.094 (7)	0.048 (10)	0.056 (8)	−0.039 (7)
O2	0.077 (6)	0.243 (16)	0.111 (10)	0.016 (7)	0.038 (6)	−0.023 (10)
O3	0.105 (9)	0.242 (16)	0.247 (18)	−0.054 (11)	0.079 (11)	−0.189 (15)
O4	0.31 (3)	0.224 (15)	0.27 (2)	0.084 (15)	0.17 (2)	0.105 (15)
O1'	0.134 (7)	0.129 (7)	0.109 (6)	0.003 (5)	0.017 (5)	−0.077 (6)
O2'	0.211 (13)	0.103 (5)	0.137 (7)	0.059 (7)	0.118 (8)	0.025 (5)
O3'	0.110 (5)	0.159 (9)	0.065 (4)	−0.011 (5)	−0.004 (4)	−0.018 (4)
O4'	0.320 (15)	0.167 (10)	0.300 (16)	−0.050 (10)	0.256 (16)	−0.056 (9)

### Geometric parameters (Å, °)

**Table:**

Mn1—N4	2.247 (3)	C5—C6	1.384 (5)
Mn1—N4^i^	2.247 (3)	C5—H5	0.9300
Mn1—N2	2.283 (3)	C6—C7	1.378 (5)
Mn1—N2^i^	2.283 (3)	C6—H6	0.9300
Mn1—N1	2.287 (3)	C7—C8	1.370 (5)
Mn1—N1^i^	2.287 (3)	C7—H7	0.9300
N1—C4	1.328 (4)	C8—C9	1.476 (5)
N1—C8	1.337 (4)	C10—C11	1.530 (5)
N2—C3	1.292 (4)	C10—H10A	0.9700
N2—C1	1.484 (5)	C10—H10B	0.9700
N3—C3	1.354 (5)	N6—C13	1.117 (10)
N3—C2	1.458 (5)	C12—C13	1.3547
N3—H3A	0.8600	C12—H12B	0.9600
N4—C9	1.287 (4)	C12—H12A	0.9600
N4—C11	1.486 (4)	C12—H12C	0.9600
N5—C9	1.332 (4)	C11—H11A	0.9700
N5—C10	1.459 (5)	C11—H11B	0.9700
N5—H5A	0.8600	Cl1—O1'	1.376 (5)
C1—C2	1.519 (5)	Cl1—O2'	1.406 (5)
C1—H1A	0.9700	Cl1—O2	1.407 (6)
C1—H1B	0.9700	Cl1—O3'	1.409 (5)
C2—H2A	0.9700	Cl1—O4	1.411 (7)
C2—H2B	0.9700	Cl1—O3	1.426 (7)
C3—C4	1.468 (5)	Cl1—O1	1.474 (7)
C4—C5	1.379 (5)	Cl1—O4'	1.505 (6)
			
N4—Mn1—N4^i^	96.14 (16)	C8—C7—H7	121.0
N4—Mn1—N2	139.28 (11)	C6—C7—H7	121.0
N4^i^—Mn1—N2	91.20 (11)	N1—C8—C7	121.8 (4)
N4—Mn1—N2^i^	91.20 (11)	N1—C8—C9	111.6 (3)
N4^i^—Mn1—N2^i^	139.28 (11)	C7—C8—C9	126.6 (4)
N2—Mn1—N2^i^	108.70 (16)	N4—C9—N5	116.9 (3)
N4—Mn1—N1	70.18 (11)	N4—C9—C8	119.4 (4)
N4^i^—Mn1—N1	87.66 (10)	N5—C9—C8	123.7 (4)
N2—Mn1—N1	70.18 (12)	N5—C10—C11	101.3 (3)
N2^i^—Mn1—N1	132.10 (10)	N5—C10—H10A	111.3
N4—Mn1—N1^i^	87.66 (10)	C11—C10—H10A	110.3
N4^i^—Mn1—N1^i^	70.18 (11)	N5—C10—H10B	112.2
N2—Mn1—N1^i^	132.10 (11)	C11—C10—H10B	112.1
N2^i^—Mn1—N1^i^	70.18 (11)	H10A—C10—H10B	109.4
N1—Mn1—N1^i^	146.97 (17)	C13—C12—H12B	114.2
C4—N1—C8	120.4 (3)	C13—C12—H12A	103.9
C4—N1—Mn1	119.4 (2)	H12B—C12—H12A	114.2
C8—N1—Mn1	119.0 (2)	C13—C12—H12C	103.9
C3—N2—C1	105.8 (3)	H12B—C12—H12C	114.2
C3—N2—Mn1	116.2 (3)	H12A—C12—H12C	105.4
C1—N2—Mn1	137.3 (2)	N6—C13—C12	180.000 (6)
C3—N3—C2	108.5 (3)	N4—C11—C10	106.3 (3)
C3—N3—H3A	125.7	N4—C11—H11A	110.9
C2—N3—H3A	125.7	C10—C11—H11A	109.5
C9—N4—C11	106.0 (3)	N4—C11—H11B	110.7
C9—N4—Mn1	118.4 (3)	C10—C11—H11B	111.2
C11—N4—Mn1	135.5 (2)	H11A—C11—H11B	108.3
C9—N5—C10	109.6 (3)	O1'—Cl1—O2'	117.7 (5)
C9—N5—H5A	125.2	O1'—Cl1—O2	88.3 (5)
C10—N5—H5A	125.2	O2'—Cl1—O2	63.0 (5)
N2—C1—C2	106.7 (3)	O1'—Cl1—O3'	112.1 (4)
N2—C1—H1A	110.4	O2'—Cl1—O3'	112.2 (5)
C2—C1—H1A	110.4	O2—Cl1—O3'	157.2 (5)
N2—C1—H1B	110.4	O1'—Cl1—O4	74.9 (8)
C2—C1—H1B	110.4	O2'—Cl1—O4	165.2 (8)
H1A—C1—H1B	108.6	O2—Cl1—O4	112.3 (7)
N3—C2—C1	102.0 (3)	O3'—Cl1—O4	66.1 (7)
N3—C2—H2A	111.4	O1'—Cl1—O3	157.0 (5)
C1—C2—H2A	111.4	O2'—Cl1—O3	61.8 (7)
N3—C2—H2B	111.4	O2—Cl1—O3	109.3 (6)
C1—C2—H2B	111.4	O3'—Cl1—O3	54.5 (6)
H2A—C2—H2B	109.2	O4—Cl1—O3	109.9 (7)
N2—C3—N3	116.7 (4)	O1'—Cl1—O1	53.6 (5)
N2—C3—C4	120.8 (3)	O2'—Cl1—O1	84.9 (6)
N3—C3—C4	122.5 (3)	O2—Cl1—O1	110.6 (6)
N1—C4—C5	121.3 (4)	O3'—Cl1—O1	90.5 (5)
N1—C4—C3	111.9 (3)	O4—Cl1—O1	109.6 (6)
C5—C4—C3	126.8 (4)	O3—Cl1—O1	104.9 (6)
C4—C5—C6	118.1 (4)	O1'—Cl1—O4'	104.9 (5)
C4—C5—H5	121.0	O2'—Cl1—O4'	106.6 (4)
C6—C5—H5	121.0	O2—Cl1—O4'	61.7 (5)
C7—C6—C5	120.4 (4)	O3'—Cl1—O4'	101.6 (5)
C7—C6—H6	119.8	O4—Cl1—O4'	60.8 (7)
C5—C6—H6	119.8	O3—Cl1—O4'	96.7 (6)
C8—C7—C6	118.0 (4)	O1—Cl1—O4'	158.3 (6)

Symmetry codes: (i) −*x*+1, *y*, −*z*+3/2.

### Hydrogen-bond geometry (Å, °)

**Table:**

*D*—H···*A*	*D*—H	H···*A*	*D*···*A*	*D*—H···*A*
N5—H5A···O2^ii^	0.86	2.50	3.237 (12)	144
N5—H5A···O3^ii^	0.86	2.25	2.942 (12)	137
N5—H5A···O2'^ii^	0.86	2.11	2.965 (8)	176
N3—H3A···O4^iii^	0.86	2.52	3.26 (2)	144
N3—H3A···O3'^iii^	0.86	2.16	3.015 (8)	178

Symmetry codes: (ii) *x*, −*y*+1, *z*+1/2; (iii) *x*+1/2, *y*+1/2, *z*+1.
